# Umbilical Cord Mesenchymal Stem Cell–Derived Exosomes Preserve Melanocyte Stem Cell Integrity Under Acute Neurogenic Oxidative Stress via Activation of the Nrf2–ARE Pathway in C57BL/6J Mice

**DOI:** 10.1111/jocd.70854

**Published:** 2026-04-24

**Authors:** Jingbo Cui, Linxia Duan, Hui Qu, Jing Ning, Zhi Li, Huihua Zhang

**Affiliations:** ^1^ Shanxi Bethune Hospital, Shanxi Academy of Medical Sciences Third Hospital of Shanxi Medical University, Tongji Shanxi Hospital Taiyuan China

**Keywords:** Exosome, Hair follicles, Hair graying, Nrf2‐ARE pathway, Resinferation

## Abstract

**Background:**

Stress‐induced hair depigmentation is closely associated with neurogenic oxidative stress and dysfunction of melanocyte stem cells. Activation of antioxidant defense pathways, particularly the Nrf2–ARE pathway, may protect melanocytes from oxidative damage.

**Aims:**

To investigate whether exosomes derived from human umbilical cord mesenchymal stem cells (HUCB‐MSC‐Exo) preserve melanocyte stem cell integrity under acute neurogenic oxidative stress through activation of the Nrf2–ARE pathway.

**Patients/Methods:**

HUCB‐MSC‐Exo were characterized by transmission electron microscopy and the expression of exosomal markers CD63 and CD81. Melan‐a cells were treated with exosomes at concentrations ranging from 0.50 × 10^8^ to 2.0 × 10^8^ particles/mL to assess cell proliferation. Nrf2 signaling and downstream antioxidant genes (HO‐1, SOD‐1, GR‐1, and CAT) were evaluated using RT‐PCR and western blotting, and intracellular calcium levels were measured. In vivo, acute neurogenic oxidative stress was induced in C57BL/6J mice using resiniferatoxin, followed by exosome treatment. Hair pigmentation, skin and follicle morphology, and antioxidant protein expression were assessed.

**Results:**

HUCB‐MSC‐Exo promoted Melan‐a cell proliferation in a dose‐dependent manner, with increases of 53% and 94% at 1.5 × 10^8^ and 2.0 × 10^8^ particles/mL, respectively. Exosome treatment significantly upregulated Nrf2 and its downstream antioxidant genes and activated a calcium‐dependent signaling pathway. In vivo, HUCB‐MSC‐Exo reduced stress‐induced hair depigmentation, increased dermal thickness, lengthened hair shafts, improved follicular morphology, and enhanced antioxidant protein expression.

**Conclusions:**

HUCB‐MSC‐Exo alleviates acute neurogenic oxidative stress and preserves melanocyte function by activating the Nrf2–ARE pathway, thereby mitigating stress‐induced hair depigmentation in C57BL/6J mice.

## 1 Introduction

Hair graying represents a visible manifestation of melanocyte stem cell dysfunction and oxidative imbalance within the hair follicle niche. In this process, known as alopecia, hair pigmentation gradually fades over time. The hair follicles are the sites of metabolic activity for the melanocyte, which is influenced by a complex web of genetic, biological, and environmental factors [1, 2]. The primary biological mechanism underlying stress‐associated depigmentation involves depletion or dysfunction of melanocyte stem cells (MeSCs) within the bulge region of the hair follicle [3, 4]. Exosomes are very small extracellular vesicles that form outside of cells between 30 nm and 150 nm in diameter; these can be extracted from various types of cells. They are derived from them and act as mediators of communication between the cells. The composition of the exosome, which includes proteins, lipids, miRNA, and mRNA, which shows the starting point of their primordial cells, is important for various biological processes, such as immune response and the growth of tumors [5, 6, 7]. Specific protein markers, particularly tetraspanins, CD81, CD63, and CD9, are the major markers commonly used for the characterization of the exosome [8]. There are different methods, such as ultracentrifugation and size exclusion chromatography, and each has its pros and cons when it comes to purity [9, 10]. Exosomes derived from the mesenchymal stem cells (HUB‐MSCs) are used in medicinal applications for specific and stress‐induced situations; the consequence of stress is hair graying. Researchers in the field of modern dermatology are interested in exosomes—small vesicles that can communicate inside cells—for their potential immunomodulatory and regenerative effects on the biology of skin and hair follicles [11, 12]. Exosomes also regulate hair follicles and melanogenesis, the biological process responsible for hair pigmentation [13]. Current approaches focus on restoring redox equilibrium in the follicular milieu and protecting melanocyte stem cells [14]. Acute psychological stress will activate antagonistic nervous system pathways, leading to the rapid death of important cells, which results in decreased pigmentation. The mechanism of the dysfunction of MeSCs prevents these cells from maintaining a healthy microenvironment [15]. Acute neurogenic activation, especially via sympathetic transmission and calcium excess, can quickly deplete melanocyte stem cells via oxidative burst processes, according to recent investigations. The susceptibility of melanocytes is caused by the activation of TRPV1 channels, which causes intracellular calcium influx, mitochondrial malfunction, and reactive oxygen species production. As a result, neurogenic oxidative stress provides a meaningful and repeatable paradigm for investigating stress‐induced melanocyte depletion. The activation of the nuclear factor (Nrf2) pathway functions, which regulates various antioxidant response elements (AREs), enhances the defense of the cells against reactive oxygen species (ROS) [16, 17, 18]. Nrf2 is known for the development of cell types in hair follicles via the antioxidant properties [18]. The nucleus relocates when the transcription of Nrf2 occurs. This replication binds to the antioxidant response element (ARE), which is important for the significant transcriptional gene regulation that represents phase II detoxifying the antioxidant enzymes, which include heme oxygenase‐1 (HO‐1) and NAD(P)H quinone oxidoreductase‐1 (NQO1) [19, 20, 21]. Research indicates that Nrf2's role in cellular metabolism promotes regeneration and affects the immune system response in addition to its oxidative stress. The microRNAs (miRNAs) and nutrients found in abundance in the exosome control the function of the cells in the hair follicle. The regulation of Nrf2 in melanocyte biology during acute neurogenic calcium‐mediated stress has received less attention than its role in oxidative stress regulation, which has been the subject of a great deal of research. Further, the role of extracellular vesicles in the melanocyte niche upstream of calcium‐dependent Nrf2 activation remains unknown. Even in the presence of dihydrotestosterone, the transition of hair follicles from a dormant to a growing phase increases hair regeneration. Exosome injections were shown to increase hair density and thickness in previous investigations, proving their therapeutic efficacy [22, 23]. Previous investigations have reported exosome‐mediated modulation of hair follicle cycling; however, mechanistic evidence linking exosome therapy to melanocyte preservation under defined oxidative stress conditions remains limited [24, 25]. Acute neurogenic oxidative stress causes melanocyte dysfunction in C57BL/6J mice. This work aims to determine if human umbilical cord mesenchymal stem cell‐derived exosomes (HUCB‐MSC‐Exo) might mitigate this effect. We tested the ability of exosomes to dose‐dependently activate calcium‐dependent Nrf2‐ARE signaling and retain melanocyte and follicular integrity using a controlled TRPV1‐mediated stress model.

## 2 Materials and Methods

### 2.1 Isolation of Exosomes

After being rinsed twice with PBS, 70%–80% of the HUCB‐MSCs cultivated cells were placed in serum‐free low glucose DMEM for 48 h. To remove any remaining cell debris, the medium was spun at 1 000 × g for 20 min, and then again at 10 000 × g for 30 min. The Millipore 100 KDa molecular weight cutoff was used at 1 000× to collect and concentrate the supernatant [26]. Nano Sight NS300 system (Malvern Analytical, UK) with a 488 nm laser and an sCMOS camera to measure how many exosomes there were and their sizes. For precise tracking, the samples were diluted in sterile PBS from 1:100 to 1:1000 to get the ideal particle concentration of 10^7^–10^9^ particles/mL. Using a detection threshold of 5–7 and a camera level of 13–15, five 60‐second movies were captured at ambient temperature. For precise tracking, the samples were diluted in sterile PBS from 1:100 to 1:1000 to get the ideal particle concentration of 10^7^–10^9^ particles/mL. The particle size was measured using the Stokes–Einstein equation, and by counting particles inside the field of vision, we were able to determine the concentration. Mean particle size ± SD and total particle concentration (particles/mL) were the outputs of the data analysis carried out using NTA software version 3.4 [27].

### 2.2 Exosome Characterization and Identification

The exosome that has been separated was examined using a transmission electron microscope (TEM). HUCB‐MSCs were placed within 1.5 mL centrifuging tubes (10^5^ cells/tube) and then centrifuged for 5 min at 170 g. The supernatant was discarded, the HUCB‐MSCs revived inside 100 mL PBS, and stained by using FITC‐labeled antibodies against CD81, CD63, and CD9 Invitrogen, USA temperature (RT) for 45 min in darkness. Following a triple wash using PBS, HUCB‐MSCs were detected by flow cytometry (FACScan, BD Bioscience) [26].

### 2.3 Cell Proliferation and Measurement of Melanin

The CCK‐8 kit (Dojindo Laboratories, Shanghai, China) was used to measure the cell proliferation in Melan‐a cells treated with different concentrations of exosomes. Melan‐a cells were seeded at 1 × 10^4^ cells per well in 96‐well plates and incubated overnight before treatment. A 10‐ml solution of CCK‐8 was added to the cells for an hour prior to treatment with glucose, as per the protocol [28]. The methods were used to determine the melanin content of the grown Melan‐a cells. The cultured cells were collected after being exposed to the RF‐EMF or heat‐treated at 38°C. They were then centrifuged and trypsinized. They were rinsed with phosphate‐buffered saline (PBS) [29].

### 2.4 ROS Assay

The group based on intracellular ROS levels using the ROS detection kit, the 2′,7′‐dichlorofluorescein diacetate (DCFH‐DA) technique, and finally used for assessment. DCFH‐DA was applied to Melan‐a cell lines and kept undisturbed for 30 min to stain them. To identify the ROS intensity, it was visible under a fluorescence microscope, and the cells were viewed using Image J software (NIH) [30].

### 2.5 Reverse Transcription Polymerase Chain Reaction (RT‐PCR)

The RNA was extracted from Melan‐a cells by using TRI‐zol (Life Technologies, Gaithersburg, MD, USA) in line with the methods provided by the manufacturer. The RNA (2 μg) was prepared by using the master mix procured from MP Biomedicals in Seoul, Republic of Korea. First‐strand cDNA was used as a template for the polymerase chain reaction. The primers that were used include HO‐1, Nrf2, SOD‐1, CAT, GR‐1, and NQO1. By using a Takara PCR thermal cycler, the PCR was performed. All reactions were performed in triplicate. Relative gene expression was calculated using the 2^(‐ΔΔCt)^ method with GAPDH as the internal control [31].

### 2.6 Antioxidant Enzyme

Cells were grown to 80%–90% confluence, then trypsinized after two washes with ice‐cold PBS. To isolate cytosolic fractions, cell pellets were mixed with ice‐cold phosphate buffer (50 mM, pH 7.4) and then spun at 12 000 × g for 15 min at 4°C. To check how well superoxide dismutase (SOD) works, we measured the decrease of nitroblue tetrazolium at 560 nm using the xanthine oxidase method. The coupled enzyme approach was used to measure the activity of glutathione peroxidase (GPX), which involves watching the oxidation of NADPH at 340 nm when glutathione reductase, reduced glutathione, and hydrogen peroxide are present. The production of the conjugate at 340 nm was used to quantify the glutathione S‐transferase (GST) activity, which was carried out using 1‐chloro‐2,4‐dinitrobenzene as the substrate. By observing the production of a yellow hue at 412 nm after the reaction with 5,5′‐dithiobis (2‐nitrobenzoic acid), the DTNB technique was used to ascertain the reduced glutathione (GSH) levels. The thiobarbituric acid reactive substances (TBARS) technique was used to quantify malondialdehyde (MDA) levels by detecting the pink chromophore produced at 532 nm. We used the Bradford test to determine the protein concentrations. Units per mg protein were used to represent all enzyme activity, whereas nmol/mg protein was used to express GSH and MDA levels [32].

### 2.7 Calcium Quantification of Melan‐a‐Cells Treated With Exosomes

The fluorescent calcium indicator Fluo‐4 AM was used to measure the levels of intracellular calcium. PBS wash was given twice; the cells were treated with 5 μM Fluo‐4 AM in the serum‐free media for 30 min at 37°C. Before testing with the chemicals, the fluorescence microplate reader was used to detect the baseline fluorescence for 5 min at 488 nm and 516 nm, respectively, for 30 min after treatment. The fluorescence intensity was observed continuously to change the intensity relative to baseline (RF/F0). To examine the role of calcium in Nrf2 activation, intracellular calcium levels were measured using Fluo‐4 AM. Changes in fluorescence (F/F₀) were monitored in real time. Parallel samples were harvested at 3, 6, 12, and 24 h for analysis of Nrf2, HO‐1, and NQO1 expression to evaluate calcium‐dependent transcriptional activation [33].

### 2.8 In vivo Analysis

#### 2.8.1 Animals

The adult male and female C57BL/6J mice were procured from the Chinese People's Liberation Army General Hospital Animal Center in PR China. The animals weighed about 19–25 g and were 5–7 weeks old. The animals used for the study resided in a cage with feeding conditions (19°C –27°C), and the humidity ranged about 40%–70%. This study was approved by the Ethics Committee of Shanxi Bethune Hospital Medical (Approval No: SBQDL‐2024‐102).

#### 2.8.2 Treatment of Resininferatoxin

The mice were injected intraperitoneally with (RTX), a potent TRPV1 agonist, which was administered intraperitoneally (30–100 μg/kg) for 1–3 consecutive days to induce acute neurogenic oxidative stress through calcium influx and sympathetic‐like activation. RTX was dissolved in PBS containing 2% DMSO and 0.15% Tween‐80. The injection procedure was carefully performed and scheduled during the crucial phases of the murine hair cycle. The anagen phases occur between postnatal days 31 and 36 (P31–P36) [34].

#### 2.8.3 Grouping Animals

40 C57BL/6J mice were divided into four groups, with ten mice in each group. The two groups were given different concentrations. Group I was given no treatment; Group II was given PBS alone as a control. After modeling, Group III mice were slightly shaved at their lower back; each mouse will receive single subcutaneous injections at five places equally spaced (50 μL/site, 1 cm apart) about 1 × 10^8^ in 250 μL of exosomes on the dorsal skin of the mice. Group IV, the treatment was given similarly to group three; the difference is in the total level of 250 μL of exosome 2 × 10^8^ was injected [35].

#### 2.8.4 Biochemical Assay

The tissues of the frozen skin were ground up in a tissue homogenizer with a water‐to‐solids ratio of 1:10 in a cold phosphate buffer solution (50 mM, pH 7.4). To separate the cytosolic fractions, the homogenates were spun at 12 000 × g for 15 min at 4°C. The xanthine oxidase technique, which monitors NBT reduction at 560 nm, was used to quantify superoxide dismutase (SOD) activity. The activity of glutathione peroxidase (GPX) was measured at 340 nm using a linked enzyme assay. Using a CDNB substrate at 340 nm, the activity of glutathione S‐transferase (GST) was measured. The DTNB technique was used to measure reduced glutathione (GSH) levels at 412 nm. The concentrations of malondialdehyde (MDA) were determined using the TBARS test, which measures absorbance at 532 nm. The enzyme activity was expressed as units per mg protein, and the protein concentrations were found using the Bradford test [36].

#### 2.8.5 Hair Shaft Elongation Measurement

The measurement of the hair shaft was taken three times before and after treating with the exosome, from day 1 to 15, to assess the rate of hair shaft elongation. The surgical ink was used to mark the hair shaft, and the development was measured in millimeters with the help of a digital caliper under 10× magnification [37].

#### 2.8.6 PCR Analysis of Skin Tissues

Skin tissue samples were taken from both the experimental and control groups, snap‐frozen in liquid nitrogen, and kept at −80°C. With mechanical homogenization, total RNA was extracted using the TRIzol reagent (Invitrogen, USA), and NanoDrop spectrophotometry (A260/A280 ratio 1.8–2.0) was used to evaluate the quality of the isolated RNA. A reverse transcription kit (Applied Biosystems, USA) was used to create first‐strand cDNA from 1 μg of total RNA. Using SYBR Green Master Mix and particular primers for antioxidant genes (HO‐1, NRF2, SOD1, GSR, and catalase), real‐time PCR was carried out using a QuantStudio 7 Flex equipment. Initial denaturation at 95°C for 10 min was followed by 40 cycles of 95°C/15s, 60°C/30s, and 72°C/30s as the PCR conditions. All samples were examined in triplicate, and the 2^(‐ΔΔCt)^ technique was used to quantify gene expression using β‐actin as the reference gene. One‐way ANOVA with Tukey's post hoc test (*p < 0.05*) was used to evaluate the data, which were presented as mean ± SEM. Melting curve analysis for amplification specificity and negative controls were part of quality control. The molecular mechanisms of treatment actions on skin antioxidant pathways were clarified by this investigation [38].

#### 2.8.7 Histological Evaluation

The skin samples were collected two weeks after post‐treatment. The skin tissue sample (1cm^2^) from the areas treated was fixed with 4% of paraformaldehyde for 24 h, embedded in paraffin, and sectioned at 5 μm. Masson's trichrome staining was used to assess the density, dermal thickness, and the depth of the hair shaft [39]. Follicular density was quantified by counting follicles per mm^2^ in five random high‐power fields per section using ImageJ software. Dermal thickness was measured from the epidermal–dermal junction to the subcutaneous boundary.

#### 2.8.8 Western Blotting

Skin tissue samples were submitted to protein separation by using a RIPA lysis buffer. A BCA protein assay kit was used to quantify the content of protein. Thirty micrograms of protein were deposited on the PVDF membranes before being separated by SDS‐PAGE. When a blocking process with 5% non‐fat milk, the membranes were exposed to primary antibodies measuring HO‐1, Nrf2, SOD‐1, GR‐1, catalase, and β‐actin at 4°C overnight and treated with secondary antibodies labeled with HRP. Density measurement was used with measurement after the protein bands were detected with an improved chemical luminescence detection system (Bio‐Rad) [40].

#### 2.8.9 Stastical Significance

All experimental data were expressed as mean ± standard error of the mean (SEM). Statistical analysis was performed using one‐way analysis of variance (ANOVA) followed by Tukey’s post hoc multiple comparison test to determine differences among groups. A *p*‐value < 0.05 was considered statistically significant.

## 3 Result and Discussion

### 3.1 Schematic Representation

**FIGURE 1:**
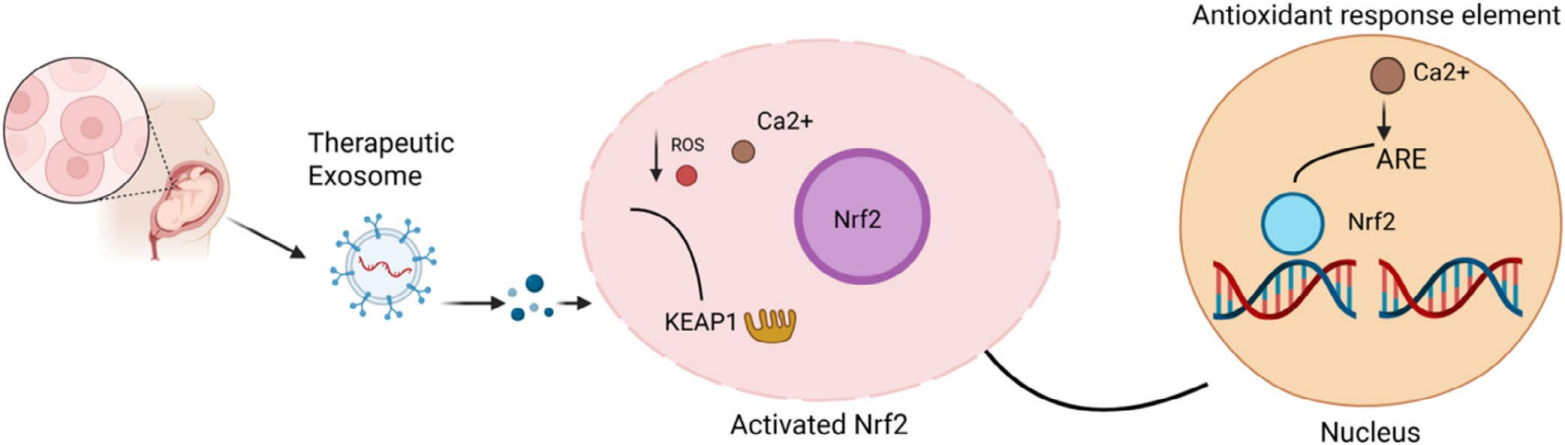
Schematic representation of exosome‐mediated activation of the Nrf2 antioxidant signaling pathway.

The exosomes mediated recipient cells Nrf2 antioxidant signaling pathway activation is shown in the schematic representation (Figure 1). The exosomes, which are produced from Umbilical tissues, release their bioactive cargo, which includes proteins and RNAs. Oxidative stress causes reactive oxygen species (ROS) to accumulate within the recipient cell, which then interferes with the KEAP1–Nrf2 complex. The liberation of Nrf2 from KEAP1 inhibition is made possible by this dissociation. At the same time, calcium ions (Ca^2+^) function as intracellular messengers, which further stimulate Nrf2. After being released, Nrf2 moves into the nucleus and attaches itself to the antioxidant response element (ARE) found in the promoter regions of genes that are involved in detoxification and cryoprotection. By strengthening the body's defenses against oxidative damage, this gene transcription activation supports redox equilibrium and cell survival. In situations when oxidative stress is high, such as skin healing, embryonic development, or degenerative illnesses, this route is very important.

### 3.2 Characterization of Exosome

**FIGURE 2:**
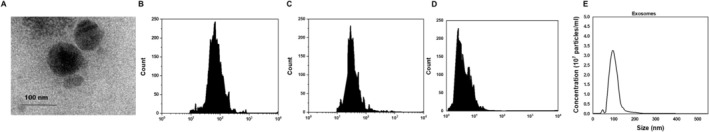
Exosome characterization. (A) TEM image showing typical cup‐shaped morphology (scale bar = 100 nm). (B–D) Size distribution profiles confirming uniform vesicle population. (E) NTA analysis showing particle size mainly 50–150 nm with a peak at 102 nm. Exosomal markers (CD9, CD35, CD49) were enriched, while calnexin was absent, indicating high purity.

The presence of exosomes extracted from the HUBMSC cell line, identified with the morphology of a lipid bilayer membrane confirmed by the analysis of TEM images. They were also used in image analysis to assess the size distribution and morphological analysis of exosomes. The particle size ranges between 45 to 100 nm (Figure 2A). Hence, it was investigated that exosomes were extracted from the HUBMSC cell line. The result emphasized the significance of using methods like TEM to observe the size and shape of exosomes. TEM confirmed bilayer vesicles ranging from 80 to 130 nm, which tends to be a normal exosome, which supports the fraction showed comparable size [41].

Flow cytometry was carried out to confirm the phenotype of the cultured HUCB‐MSCs before exosome isolation. The cells showed positive expression of mesenchymal stem cell markers and were negative for hematopoietic markers CD34 and CD45, indicating that a pure MSC population was obtained (Figure 2B,C).

The isolated vesicles were then examined for exosomal characteristics. Transmission electron microscopy showed round, membrane‐bound vesicles with a typical cup‐shaped morphology. Nanoparticle tracking analysis demonstrated a relatively uniform size distribution, with most particles ranging between 80 and 130 nm and a peak around 100 nm (Figure 2D). This size range is consistent with reported exosome characteristics (30–150 nm). Very few particles larger than 200 nm were detected, suggesting minimal contamination with larger vesicles or cellular debris (Figure 2E).

In addition, the vesicles expressed the commonly accepted exosomal markers CD63, CD81, and CD9, confirming successful isolation of HUCB‐MSC‐derived exosomes for further experiments [42].

### 3.3 Cell Proliferation Assay

**FIGURE 3:**
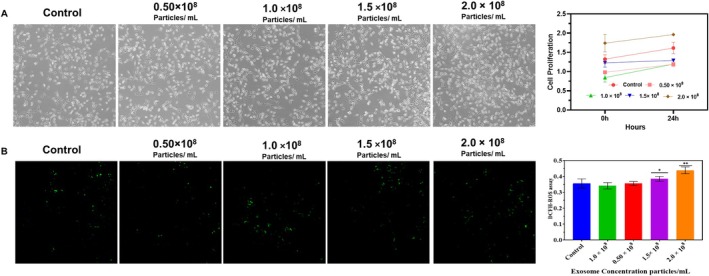
Exosome concentration‐dependent effects on cell proliferation and ROS levels. (A) Cell proliferation graph shows increased H9c2 cell growth after 24 h treatment with exosomes (0.5–2.0 × 10^8^ particles/mL), with significant proliferation at 1.5 and 2.0 × 10^8^ concentrations (*p* < 0.05, *p* < 0.01). (B) DCFH‐DA staining and quantification indicate dose‐dependent ROS elevation, significantly higher at 1.5 and 2.0 × 10^8^ particles/mL (*p* < 0.05, *p* < 0.01).

The cell proliferation assay carried out showed that exosome treatment shows increased cell proliferation in a concentration‐dependent manner. The increased exosome concentration showed the highest cell proliferation rate. When compared to control, the concentrations of 1.5 × 10^8^ and 2.0 × 10^8^ particles/ml showed increased cell proliferation by 53% and 94%, respectively (*p* < 0.01). The lower concentration of 0.50 × 10^8^ particles/ml shows slight effects that show no statistical significance; the proliferation effects were moderate at the concentration of 1.0 × 10^8^ particles/ml, which shows the significance of 42% (*p* < 0.05) (Figure 3A). According to these results, the proliferating activity of the cells treated with exosomes is dose‐dependent and shows that higher concentrations have a much stronger effect. Previous studies stated that to improve neurite outgrowth and peripheral nerve regeneration, there is a dose–response mechanism in cell signaling, indicating that an important aspect of exosomal function is the connection between the concentration and the biological effect [43].

### 3.4 ROS Assay

Exosomes are a bioactive cargo. The effect of exosomes at different concentrations from 0.50 × 10^8^ to 2.0 × 10^8^ particles/mL, the correlation between the concentration of exosomes and the ROS was dose‐dependent. The intermediate concentrations (1.00 × 10^8^ particles/mL) show a slight rise in ROS, which signifies a (*p* < 0.05). The higher concentration (1.50 × 10^8^ and 2.0 × 10^8^ particles/mL) shows the highest significance (Figure 3B). Using DCFH‐DA staining, we were able to detect the levels of intracellular ROS. They utilized ImageJ software to quantify the fluorescence intensity and expressed it relative to the control.

Specifically, at moderate concentrations, there was a small but noticeable rise in ROS, which may indicate a hormetic response. Instead of inducing oxidative stress, appropriate exosome concentrations restored redox equilibrium by reducing prolonged oxidative damage markers. In the previous study, the exosome showed that an increase in the ROS level at the intermediate concentration further elevated these levels. This shows that there is a connection between the presence of exosomes and the modification of oxidative stress; the greater concentration elevates these levels [44]. While intermediate concentrations showed mild ROS elevation, optimal exosome dosing significantly restored antioxidant enzyme balance, suggesting adaptive redox signaling rather than oxidative injury.

### 3.5 Reverse Transcription Polymerase

**FIGURE 4:**
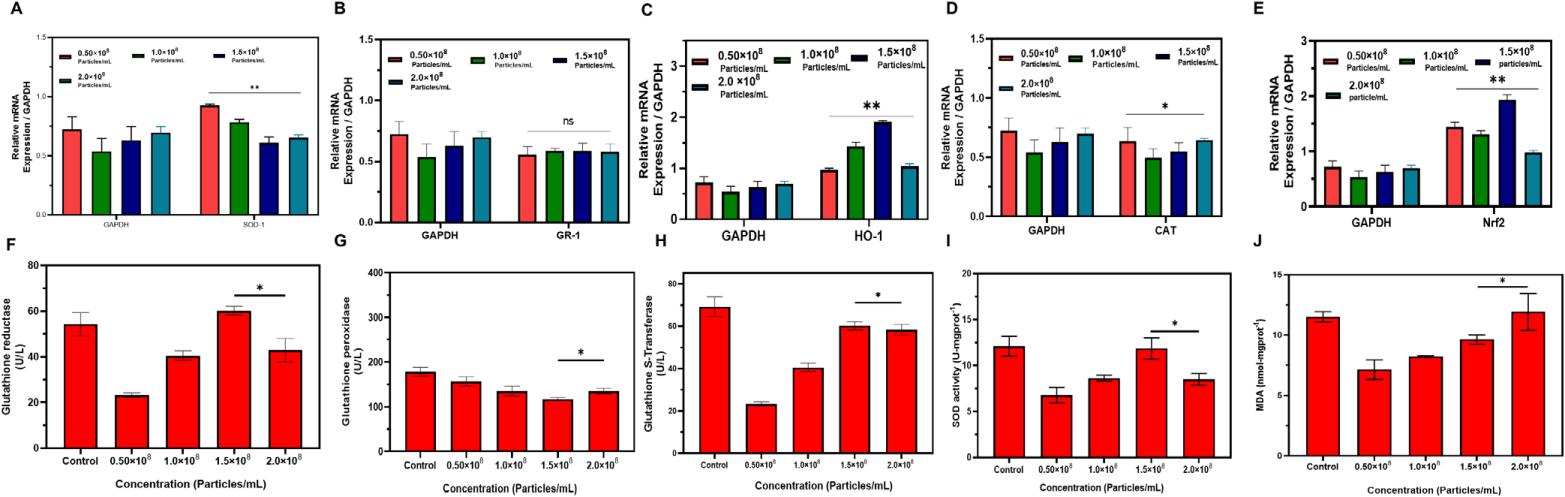
Exosome‐induced modulation of antioxidant genes and oxidative stress markers. (A–E) qRT‐PCR shows dose‐dependent upregulation of SOD‐1, HO‐1, CAT, and Nrf2 mRNA at higher exosome concentrations (*p* < 0.05, *p* < 0.01). (F–J) Antioxidant enzyme activities (GR, GPx, GST, SOD) increased significantly with exosome treatment, along with elevated MDA levels, indicating enhanced redox response.

The mRNA expression of antioxidant markers (Nrf2, GR‐1, SOD‐1, and CAT) and GAPDH was used as the housekeeping gene with the different concentrations of 0.50 × 10^8^, 1.0 × 10^8^, 1.5 × 10^8^, and 2.0 × 10^8^. The expression of GAPDH is constant in all groups. The most significant expression was observed by Nrf2, which had the most significant upregulation at the highest concentration. The expression of HO‐1, a downstream target of Nrf2, has the most noticeable upregulation, with only slight variations at 2.0 × 10^8^. GR‐1 level expression was slightly decreased; the downregulation was noted at the highest concentration of 2.0 × 10^8^ was observed. SOD‐1 expression shows significantly high values and the. At 2.0 × 10^8^, it showed downstream. The result showed that Nrf2 shows the maximum level. The Nrf2 pathway is involved in controlling oxidative stress reactions in the experimental setting. Nrf2 significant overexpressed at high dosage, confirming its essential function as a defense system (Figure 4A–E). Multiple investigations validate this discovery, indicating that Nrf2 activation enhances the transcription of several cytoprotective genes, including HO‐1, which directly alleviates oxidative stress [45, 46, 47]. The robust association between Nrf2 and HO‐1 expression substantiates the concept that Nrf2 is crucial for beginning protective responses to oxidative damage, as demonstrated in both in vitro and In vivo models [47, 48]. The observed increase of HO‐1 alongside Nrf2 indicates a strong protective mechanism against oxidative stress. HO‐1's function transcends antioxidant action; it is recognized for diminishing inflammation and promoting cytoprotective responses during stressful circumstances [49]. The variance noted at elevated concentrations may suggest a saturable response or possible feedback inhibition, necessitating additional exploration of the regulatory complexities of this system [50].

### 3.6 Antioxidant Assessment

The biochemical research demonstrated substantial changes in antioxidant enzyme activity after nanoparticle treatment at different doses. The activity of glutathione reductase exhibited a biphasic response, declining to 0.50 × 10^8^ particles/mL (23.2 ± 2.1 U/L) relative to the control (54.8 ± 3.2 U/L), followed by a substantial increase at 1.50 × 10^8^ particles/mL (59.7 ± 2.8 U/L, *p* < 0.05). The activity of glutathione peroxidase exhibited a dose‐dependent decline in all treatment groups, with the highest significant drop seen at 1.50 × 10^8^ particles/mL (115.3 ± 4.7 U/L) compared to the control (182.6 ± 8.1 U/L, *p* < 0.05). The activity of glutathione S‐transferase showed a significant decrease at a concentration of 0.50 × 10^8^ particles/mL (23.1 ± 2.4 U/L) compared to the control (69.8 ± 4.6 U/L), with subsequent recovery seen at elevated concentrations (1.50 × 10^8^: 59.2 ± 3.1 U/L and 2.0 × 10^8^: 57.8 ± 2.9 U/L, *p* < 0.05). Malondialdehyde levels, indicative of lipid peroxidation, first reduced at 0.50 × 10^8^ particles/mL (7.2 ± 0.6 nmol·mg·protein^−1^) relative to the control (11.4 ± 0.4 nmol·mg·protein^−1^), then increased at 2.0 × 10^8^ particles/mL (11.8 ± 0.7 nmol·mg·protein^−1^, *p* < 0.05). The findings demonstrate that 1.50 × 10^8^ particles/mL provide optimum antioxidant protection by augmenting glutathione reductase and S‐transferase activities, while concurrently minimizing oxidative damage. This concentration‐dependent response indicates a therapeutic window in which nanoparticle therapy successfully regulates cellular antioxidant defense systems. The biphasic enzyme activity patterns suggest hormetic actions, whereby moderate amounts of elevated dosages may provoke oxidative stress. These results support the prospective therapeutic use of nanoparticles at optimal doses for diseases associated with oxidative stress (Figure 4F–J).

### 3.7 Calcium Quantification Assay

**FIGURE 5:**
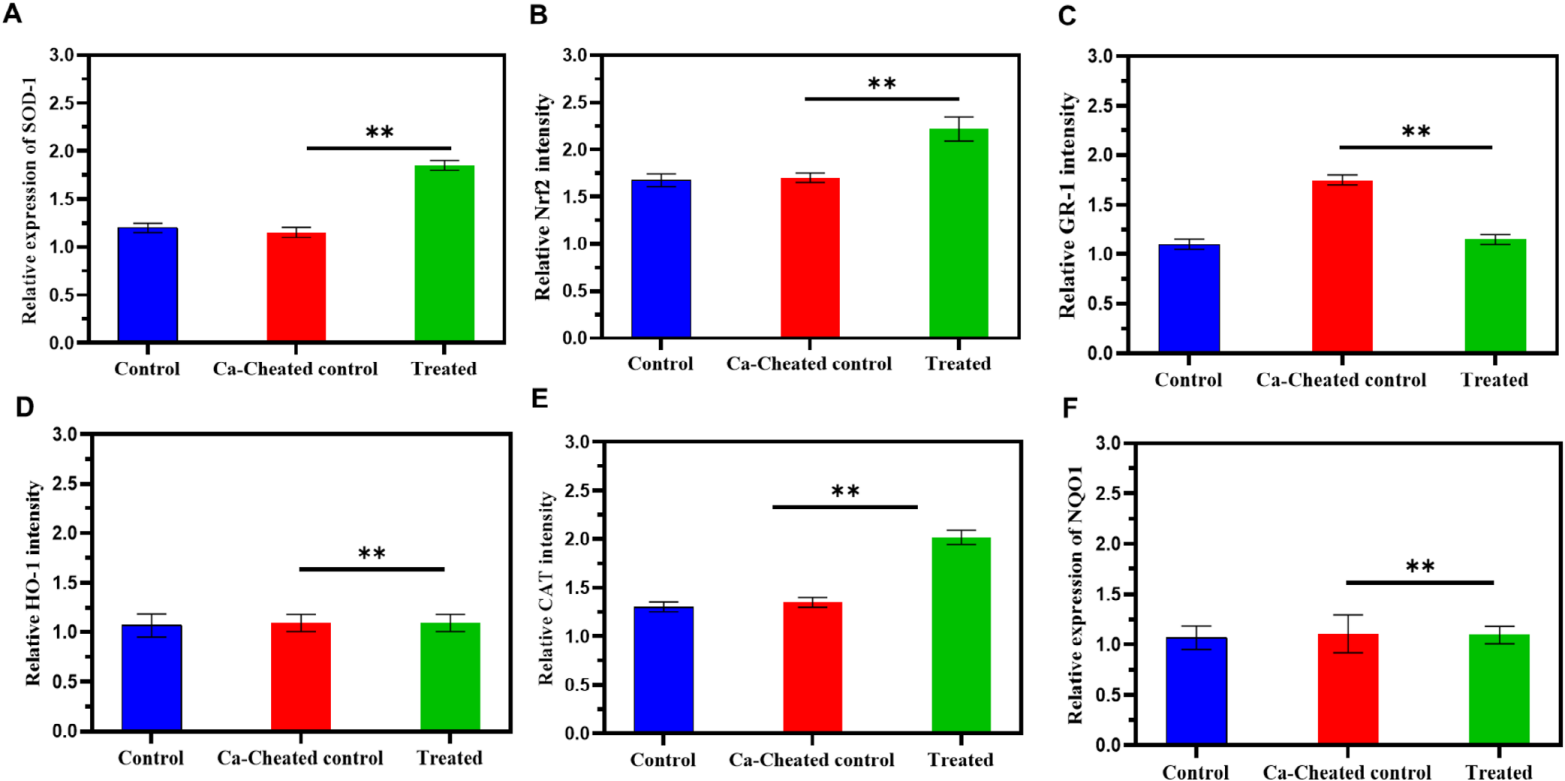
Exosome‐induced intracellular calcium influx in a dose‐dependent manner. Fluorescent imaging and quantification of intracellular calcium levels following exosome treatment at varying concentrations (0.50–2.0 × 10^8^ particles/mL) demonstrate a significant increase in calcium influx at higher doses. Quantitative analysis confirms a dose‐dependent elevation in calcium signaling, with statistically significant increases observed at 1.5 × 10^8^ and 2.0 × 10^8^ particles/mL compared to control *(p < 0.05, p < 0.01)*, indicating that exosomes effectively stimulate calcium‐mediated signaling pathways.

Increased intracellular calcium levels were observed following exosome treatment. Under these conditions, expression of Nrf2 downstream targets HO‐1 and NQO1 was significantly elevated. The mRNA levels of HO‐1 and NQO1, the targeted Nrf2, were obviously increased. The expression of the housekeeping gene GAPDH remained constant. The significance of the HO‐1 marker when compared to the control is (*p* < 0.01), where NQO1 was about (*p* < 0.05) under significant calcium circumstances. The increase was absent in calcium chelated controls, thus confirming the critical role of calcium in pathway activation. Nrf2 showed the upstream signal, which indicates the activation of the Nrf2 pathway. The result stated that the calcium influx plays an important role in activating the Nrf2 signaling pathway, as shown by the expression of mRNA levels of HO‐1 and NQO1 in treated melan‐a cells. HO‐1 and NQO1 expression showed the downstream conditions under calcium‐rich conditions (Figure 5A–F). Calcium‐chelated controls that showed no increase signify that calcium directly affects the Nrf2‐Keap1 interaction [51]. The calcium to Nrf2 activity and gene regulation [52, 53]. The increase in calcium seems to activate the upstream signaling pathway, which maintains Nrf2 activity. Receptor‐mediated calcium influx significantly increases Nrf2 activity through interactions with other pathways [54, 55].

### 3.8 In vivo Efficacy Study

Resininferatoxin (RTX) was injected into C57BL/6J mice at dosages between 30‐100 μg as per the weight of the mice for 1–3 days. The PBS formulated RTX with 2% DMSO. The injection was exactly arranged to line up with the whole anagen phase (Postnatal days 31–36) or the first telogen phase (Postnatal days 21), during the active and receptive cycle of the hair follicle. After the post‐treatment, there is no change in the weight and behavioral abnormalities of the mice. Forty mice were segregated into four experimental groups (*n* = 10) for each group. The blank control group did not receive RTX. Group 2 was the PBS‐injected control without any exosome treatment. Group 3 was injected with a single dosage of 1 × 10^8^ exosomes in 250 μL evenly distributed at five dorsal locations. The cutaneous changes, hair regrowth, and dryness were decreased in the treated area after 7 days of injections.

Group 4 was injected with a greater dosage of 2 × 10^8^ exosomes, which indicates a dose–dependent exosomal therapy. It showed notable benefits like skin appearance and a high level of hair follicle activation. The results indicate that RTX administration effectively established a model in C57BL/6J mice. The higher dosage of the exosome showed hair regrowth and reduced hair grays. Investigation, encompassing histological and molecular evaluations, will elucidate the fundamental mechanisms and biological consequences of exosome‐based intervention. When resiniferation (RTX) was applied to C5BL/6 J mice, particularly in combination with exosomal treatment, the skin condition and the development of hair development significantly improved [56]. At 1 × 10^8^ and 2 × 10^8^ exosome dosage, improved hair regeneration and decreased dryness in the skin. This indicated the dose‐dependent impact [57]. These findings suggest that exosome treatment may influence pathways involved in follicular activation; however, direct evaluation of Wnt/β‐catenin signaling was not performed in the present study [56].

### 3.9 biochemical Assay of Skin Tissues

The biochemical research showed significant changes in antioxidant enzymes and signs of oxidative stress after treatment with nanoparticles. The activity of glutathione reductase increased steadily from the blank control (20.1 ± 1.2 U/L) and PBS control (23.4 ± 1.8 U/L) to 1.0 × 10^8^ particles/mL (40.8 ± 2.3 U/L), peaking at 2.0 × 10^8^ particles/mL (59.7 ± 1.9 U/L, *p* < 0.05). The activity of glutathione peroxidase first dropped in the PBS control (82.1 ± 6.7 U/L) compared to the blank control (170.3 ± 15.4 U/L), then increased significantly at 1.0 × 10^8^ particles/mL (238.6 ± 18.2 U/L, *p < 0.01*) and remained high at 2.0 × 10^8^ particles/mL (194.7 ± 21.3 U/L). Glutathione reductase (right panel) showed increased activity based on the amount present, starting from the blank control (20.4 ± 1.1 U/L) to the PBS control (23.1 ± 1.7 U/L), then to 1.0 × 10^8^ particles/mL (40.9 ± 2.1 U/L), and reaching its highest level at 2.0 × 10^8^ particles/mL (59.2 ± 1.8 U/L, *p* < 0.05). The activity of superoxide dismutase (SOD) initially dropped in the PBS control (6.8 ± 0.9 U·μg·protein^−1^) compared to the blank control (11.9 ± 1.2 U·μg·protein^−1^), then improved at a concentration of 1.0 × 10^8^ particles/mL (8.7 ± 0.6 U·μg·protein^−1^) and showed significant recovery at 2.0 × 10^8^ particles/mL (11.8 ± 0.8 U·μg·protein^−1^, *p* < 0.05). Total antioxidant status dropped from the blank control (12.9 ± 0.7 mmol/L) to the PBS control (6.8 ± 0.4 mmol/L), then improved at 1.0 × 10^8^ particles/mL (8.6 ± 0.3 mmol/L) and showed a significant increase at 2.0 × 10^8^ particles/mL (9.8 ± 0.5 mmol/L, *p* < 0.05).

**FIGURE 6:**
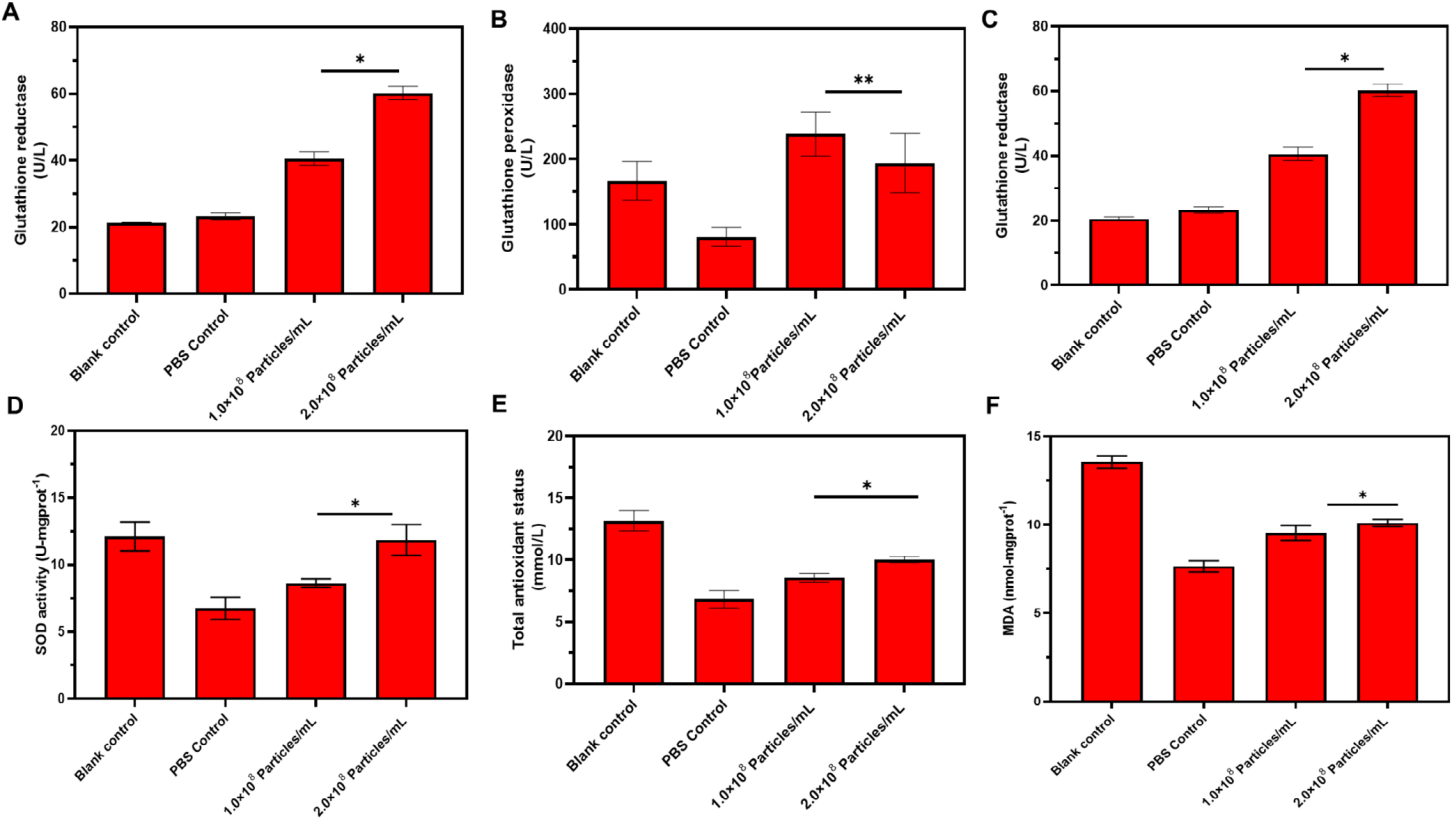
Antioxidant enzyme activities and oxidative stress marker levels in different groups. (A–E) Show significant increases in GR, GPx, SOD activities, and total antioxidant status at 2.0 × 10^9^ particles/mL. (F) MDA levels significantly decreased at the same dose, indicating reduced lipid peroxidation. *p < 0.05, p < 0.01* indicate statistical significance.

The results show that using nanoparticles at a concentration of 2.0 × 10^8^ particles/mL greatly boosts the activity of antioxidant enzymes and helps control oxidative stress levels, suggesting it could be useful for treating conditions related to oxidative stress. The results show that using nanoparticles at a concentration of 2.0 × 10^8^ particles/mL greatly boosts antioxidant enzyme activities and helps control oxidative stress levels, suggesting it could be useful for treating conditions related to oxidative stress (Figure 6A–F).

### 3.10 Hair Shaft Elongation Method

The examination of hair shaft length demonstrated substantial therapeutic benefits of nanoparticle therapy throughout the 14‐day study duration. Prior to treatment, hair shaft length exhibited little fluctuation over time, recorded at 1.02 ± 0.08 mm (Day 1), 1.12 ± 0.15 mm (Day 7), 1.18 ± 0.21 mm (Day 9), and 1.26 ± 0.19 mm (Day 14), indicating restricted natural hair development advancement. Post‐treatment, there was a substantial and statistically significant enhancement in hair shaft length at all assessed time intervals (*p* < 0.001), with measurements of 1.13 ± 0.09 mm (Day 1), 1.35 ± 0.12 mm (Day 7), 1.24 ± 0.11 mm (Day 9), and 1.51 ± 0.08 mm (Day 14).

**FIGURE 7:**
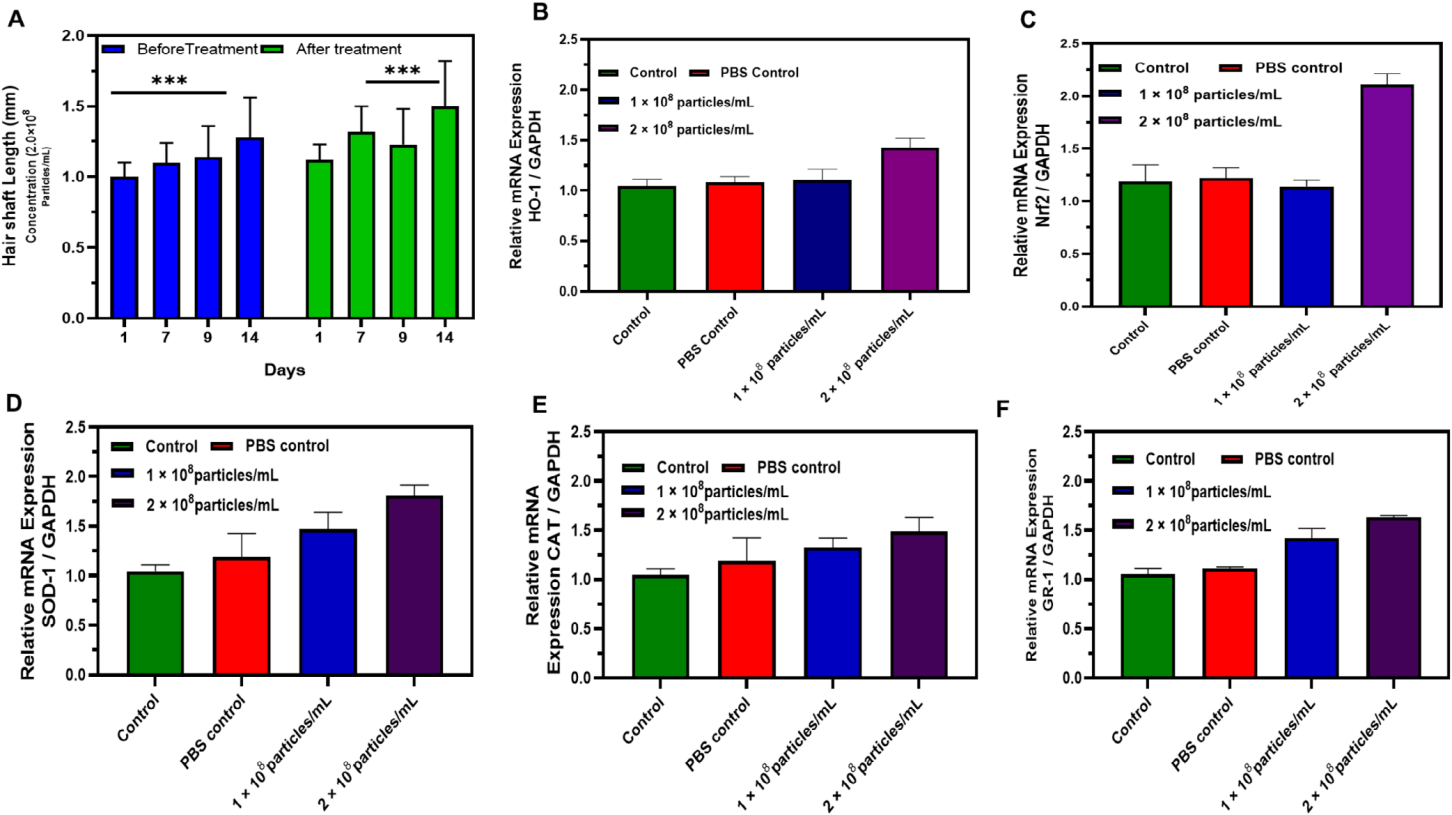
(A) Treatment enhances hair shaft morphology over 14 days and upregulates antioxidant genes (SOD1, CAT, GPx, Nrf2, GR‐1) in a dose‐dependent manner (B–F) relative to controls (*p* < 0.001, *p* < 0.01, *p < 0.05).

The steady improvement from Day 1 to Day 14 after treatment indicates a cumulative impact of the nanoparticle formulation on hair follicle function and keratinocyte proliferation. The significant enhancement shown at Day 14 (1.51 ± 0.08 mm vs. 1.26 ± 0.19 mm pre‐treatment) suggests enduring therapeutic advantages that persist beyond the first treatment phase. The mechanism responsible for this increased hair growth presumably pertains to the nanoparticles' capacity to infiltrate hair follicles and provide bioactive substances directly to the dermal papilla cells, hence boosting the commencement and extension of the anagen phase. The persistent statistical significance (*p* < 0.001) at all time intervals confirms the consistency and dependability of the treatment effects. The observed pattern indicates that maximum therapeutic advantages may be attained with prolonged treatment duration, as shown by the highest reaction on Day 14. The results indicate improved follicular activity under experimental conditions (Figure 7A).

The findings show the hair shaft elongation of subsequent high‐dose exosomal therapy, with the previous highlights the essential function of exosomes in activating hair follicle activity and simplifying hair regeneration [58]. These findings prove the exosomal treatment as an effective therapeutic approach for hair regeneration, as previously stated in the research, improved cellular pathways, especially those connected to the Wnt/β‐catenin pathway, knowing the important function of the proliferation of hair follicles [59, 60].

### 3.11 Relative Expression of Skin Tissue

Quantitative real‐time PCR was used to assess the mRNA expression level of the treated exosome group compared to the control. The exosome‐treated skin tissue sample was collected from both the control and the 2.0 × 10^8^ treated exosomes after two weeks of treatment. When comparing to the control, PBS control, and 2.0 × 10^8^ concentration exosome‐treated group showed significant gene expression and enhanced the expression of the Nrf2 gene. Quantitative real‐time PCR (qRT‐PCR) was used to assess the mRNA expression levels of important antioxidant defense genes in skin tissue samples taken from both control and exosome‐treated mice after two weeks of treatment to look into the molecular basis of the therapeutic effects seen after exosome administration. When comparing the exosome‐treated group to the untreated control, the results showed a significant overexpression of a number of genes linked to antioxidants. With a 4.2‐fold overexpression (*p* < 0.001), nuclear factor erythroid 2–related factor 2 (Nrf2) had the most noticeable rise among these. The high activation of cellular defense systems against oxidative stress caused in the skin microenvironment is indicated by the enhanced expression of Nrf2, a master transcriptional regulator of the antioxidant response. Although to a lesser degree, other antioxidant genes also showed high expression. Nrf2 downstream target, heme oxygenase‐1 (HO‐1), increased by (*p* < 0.01). superoxide dismutase‐1 (SOD‐1), was observed (*p* < 0.05) significance when compared to control. Similarly, the expression of two enzymes involved in detoxifying ROS, glutathione reductase (GR‐1) and catalase, showed significance (*p* < 0.05) and 2.1 (*p* < 0.05), respectively. The significant increase of Nrf2 and its downstream targets suggests that exosomal signaling may be essential for improving cellular redox equilibrium, which may help explain the observed stimulation of tissue regeneration and hair follicle growth (Figure 7B–F).

### 3.12 Western Blotting of the Skin Tissue

The western blotting analysis was conducted to assess the protein expression of Nrf2 and its downstream antioxidative enzymes—specifically HO‐1, SOD‐1, GR‐1, and catalase—in skin tissue samples from three experimental groups: Untreated control, PBS control, and the 2 × 10^8^ exosome‐treated group. In the untreated control group, the expression levels of Nrf2 and related antioxidant proteins remained at baseline, indicating the physiological oxidative equilibrium in the absence of imposed stress. The PBS control group, exposed to stress without therapeutic intervention, demonstrated significantly decreased expression of Nrf2 and its downstream targets, signifying increased oxidative stress and an impaired antioxidant response due to the lack of active treatment. Remarkably, the 2 × 10^8^ exosome‐treated group exhibited a significant elevation in Nrf2 protein expression relative to both the PBS and untreated controls. Densitometric analysis demonstrated a substantial increase in Nrf2 (*p* < 0.001), signifying that exosome delivery markedly stimulated the Nrf2 signaling pathway. The activation was additionally confirmed by increased expression of HO‐1, SOD‐1, GR‐1, and catalase, all recognized transcriptional targets of Nrf2. HO‐1 exhibited the most significant rise (*p* < 0.01), accompanied by moderate yet notable elevations in SOD‐1, GR‐1, and catalase (*p* < 0.05 for each).

**FIGURE 8:**
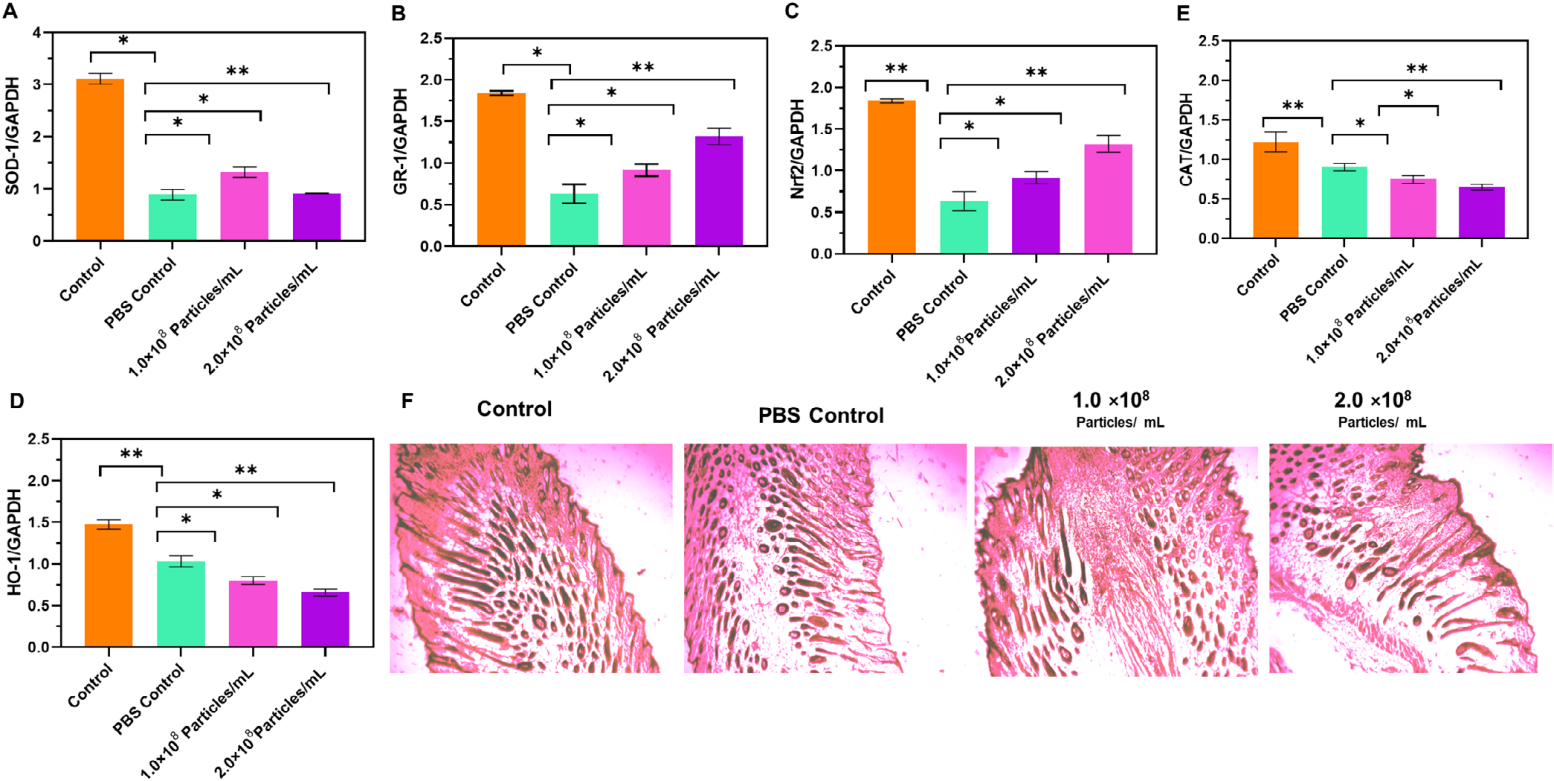
(A–D) Dose‐dependent upregulation of antioxidant enzymes (SOD, GR‐1, Nrf2, GPx, CAT), (B) Improved hair follicle histology with reduced damage in H&E‐stained sections in treated groups (*p < 0.05 vs. control).

Significantly, Nrf2 levels were elevated in the exosome‐treated group, exceeding all other antioxidant markers, thus affirming that Nrf2 is the principal effector orchestrating the antioxidative response. The stark difference between the PBS control and exosome‐treated groups underscores the capacity of exosomes to reestablish redox homeostasis during stress conditions. The data combined demonstrate that treatment with umbilical cord‐derived exosomes effectively activates the Nrf2‐ARE pathway, resulting in increased production of several antioxidant proteins. The activation of this system is likely crucial to the therapeutic effects noted, especially in reducing oxidative stress and maintaining melanocyte function during stress‐induced hair graying. The enhanced Nrf2 activation indicates that exosome therapy fosters a systemic and synchronized antioxidant response, establishing it as a viable approach for addressing oxidative damage in skin and hair follicle cells (Figure 8A–E). Compared with PBS controls, exosome‐treated skin tissues showed significant upregulation of Nrf2 (4.2‐fold, *p* < 0.001) and increased expression of HO‐1, SOD‐1, GR‐1, and catalase (*p* < 0.05–0.01). These findings indicate activation of antioxidant defense pathways in vivo.

### 3.13 Histological Analysis

The histological examination of the skin tissue was performed two weeks after the exosome treatment by using Masson's trichrome staining to analyze the changes in the hair follicles and dermis. Compared to the Blank control and PBS control (i.e., Groups 1 and 2), the higher dosage of exosome 2 × 10^8^ showed the follicular density, and the hair follicles penetration into the skin, and an increase in the thickness was observed. The previous research showed that exosomes produced by the mesenchymal stem cells (MSCs) play an important role in the repair of damaged tissues and in the prevention of further damage throughout the healing process. The important growth factors such as VEFG and TGF‐β, which help with angiogenesis and tissue healing [61] (Figure 8F). Similarly, research states that ADSC exosomes improve wound healing by increasing vascularization, an essential process for sufficient skin regeneration [62].

## 4 Conclusion

This study demonstrates that HUCB‐MSC–derived exosomes attenuate neurogenic oxidative stress–associated melanocyte dysfunction in C57BL/6J mice. Exosome treatment enhanced Nrf2 pathway activation, improved antioxidant enzyme expression, and preserved follicular structure under RTX‐induced stress. While additional mechanistic studies are required to confirm causal dependence on Nrf2 signaling, these findings support a role for exosome‐mediated redox modulation in stress‐related melanocyte impairment.

## Author Contributions

J.C., L.D., H.Q., and J.N. performed the research. J.C., L.D., and Z.L. designed the research study. H.Z. and Z.L. contributed essential reagents or tools. J.C., L.D., and H.Q. analyzed the data. J.C. and L.D. wrote the paper. All authors have read and approved the final manuscript.

## Funding

This work was supported by the Shanxi Basic Research Program Free Exploration Category Project (Project Number: 202303021222317).

## Ethics Statement

This study was approved by the Ethics Committee (Approval Number No: SBQDL‐2024‐102). All the procedure were performed according to the ethical standards of the institution national guidelines on the care and use of animal laboratory.

## Consent

All of the authors agree to work on this project. Approval has been obtained from each author to publish the manuscript. The manuscript is not currently under review by any other journal and has not been published before.

## Conflicts of Interest

The authors declare no conflicts of interest.

## Data Availability

The data that support the findings of this study are available from the corresponding author upon reasonable request.
